# Effects of In Vitro Digestion on Anti-α-Amylase and Cytotoxic Potentials of *Sargassum* spp.

**DOI:** 10.3390/molecules27072307

**Published:** 2022-04-02

**Authors:** Sovannary Un, Nguyen Van Quan, La Hoang Anh, Vu Quang Lam, Akiyoshi Takami, Tran Dang Khanh, Tran Dang Xuan

**Affiliations:** 1 Transdisciplinary Science and Engineering Program, Graduate School of Advanced Science and Engineering, Hiroshima University, Hiroshima 739-8529, Japan; unnsovannary@gmail.com (S.U.); hoanganh6920@gmail.com (L.H.A.); 2 Division of Hematology, Department of Internal Medicine, Aichi Medical University School of Medicine, Nagakute 480-1195, Japan; quanglamvu1991@gmail.com (V.Q.L.); takami-knz@umin.ac.jp (A.T.); 3 Agricultural Genetics Institute, Pham Van Dong Street, Hanoi 122000, Vietnam; khanhkonkuk@gmail.com; 4 Center for Agricultural Innovation, Vietnam National University of Agriculture, Hanoi 131000, Vietnam

**Keywords:** *Sargassum* spp., α-amylase inhibitor, anti-diabetic, cytotoxicity, U266 cell line, multiple myeloma

## Abstract

This is the first study to examine the effects of in vitro digestion on biological activities of *Sargassum* spp., a broadly known brown seaweed for therapeutic potential. Three fractions (F1–F3) were obtained from hexane extract by column chromatography. Under in vitro simulated digestion, the anti-α-amylase capacity of F1 in oral and intestinal phases increases, while it significantly decreases in the gastric phase. The α-amylase inhibition of F2 promotes throughout all digestive stages while the activity of F3 significantly reduces. The cytotoxic activity of F1 against U266 cell-line accelerates over the oral, gastric, and intestinal stages. The fractions F2 and F3 exhibited the declined cytotoxic potentialities in oral and gastric phases, but they were strengthened under intestinal condition. Palmitic acid and fucosterol may play an active role in antidiabetic and cytotoxic activity against multiple myeloma U266 cell line of *Sargassum* spp. However, the involvement of other phytochemicals in the seaweed should be further investigated.

## 1. Introduction

Diabetes mellitus is one of the most serious human chronic metabolic disorders. In 2019, over 460 million people have been estimated to have diabetes. This number is possibly projected to be 578 million by 2030 and 700 million by 2045 [1]. Among diabetes mellitus, type 2 diabetes is the most common chronic metabolic disorder. The disease occurs when the pancreas does not produce enough insulin or when the body’s cells are insulin resistant, leading to hyperglycemia after meals [2]. Among sugar hydrolyzed enzymes, α-amylase is a crucial digestive enzyme that catalyzes the formation of glucose molecules from polysaccharides [3]. It has been proven that inhibiting α-amylase activity can be a practical approach to prevent and control hyperglycemia in diabetic patients [4]. More worrisome than diabetes, cancer is another severe chronic disease that requires complicated and costly therapies. Among cancer cases, multiple myeloma or myeloma is a formation of malignant tumors within plasma cells, which is characterized as the second most popular blood cancer after leukemia [5]. U266 is derived from a human multiple myeloma cell line with IgE secreting myeloma [6], which has been commonly used as an experimental model for hematological cancer studies. The inhibition of the U266 cell line will lead to a potential solution for preventing multiple myeloma. Moreover, the correlation between hyperglycemia and cancer was reported [7]. Hyperglycemia, hyperinsulinemia, or using drugs for diabetic patients might affect all tissues and could lead to cancer initiation or progression in any organs [8,9]. The biological mechanism between diabetes and cancer has not been fully explained and requires further investigation [10]. However, there is evidence that diabetes patients treated with insulin sensitizers including metformin and thiazolidinediones can develop prostate [11] and breast cancers [12]. Compared to metformin, diabetes treatment with insulin or insulin secretagogues has a higher risk of getting cancer [13]. On the other hand, cancer treatment caused elevated glucose in the bloodstream [14]. As a result, patients receiving diabetic treatment may be at potential risk of getting cancer and vice versa. Therefore, the discovery of novel and effective medicines which can simultaneously prevent the risks of diabetes and cancer is honestly necessary for future treatments of such chronic diseases.

Recently, the research on plant-based medicines has broadened globally. Many plants have been investigated for their pharmaceutical properties. Among them, *Sargassum* (brown seaweed) is distributed widely in tropical and subtropical areas [15] and has been used for foods and folk remedies in many countries [16]. *Sargassum* seaweeds have antidiabetic [17], antioxidant and anticancer [18,19], anti-stress [20], antimicrobial [21], antiviral [22], and anti-inflammatory [23] properties. The *Sargassum* species has indicated potential anti-diabetic activities via α-amylase inhibitory activity capacity [24,25]. On the other hand, the anticancer activities of *Sargassum* have been reported by various studies, of which fucoidan is the most outstanding polysaccharide exerting potent anticancer activity [26]. This natural source is promising for the development of alternative medicines and therapeutics which can prevent human chronic disorders, especially diabetes and cancer.

Besides the biological effects, the dose of phytochemicals consumption into the body has gained more attention. It has been reported that the biological activities of many functional compounds are affected over the digestive system due to the transformation of original compounds into other components with different bioaccessibility and bioactivity [27]. Investigations on the variation of phytochemicals during digestion are necessary to recognize and confirm their bioaccessibility, bioavailability, biological activity, and pharmacological properties [28]. However, the implementation of experiments on human or animal models is expensive, challenging, and required ethical constraints, thus, in vitro gastrointestinal digestion models simulated human digestive process have been introduced and widely applied in the novel natural product-based drug research recently. The models are designed mimicking the physiological conditions of a human digestive system consisting of the mouth, stomach, and small intestine stages. The advantages of in vitro digestive models include their low cost, usefulness, flexibility, and that there are no ethical concerns regarding clinical trials to investigate structural modifications, digestibility, and the release of food elements [29]. A thorough understanding of the effects of human digestion on the bioaccessibility of potential candidate substances is vital to developing an effective therapy for treating chronic illnesses [30].

To date, no research about the effect of in vitro digestion on anti-diabetic and antitumor activities of *Sargassum* spp. has been conducted. Therefore, the present study was carried out to isolate the bioactive components from *Sargassum* spp. and investigate the influence of in vitro digestion on their biological activities via enzymatic and cytotoxic assays. A bio-guided assay on fractions (F1–F3) from hexane extract of *Sargassum* spp. was conducted to examine anti-α-amylase and cytotoxic activities on human multiple myeloma U266 cell line which were then evaluated via simulated in vitro digestive model.

## 2. Results

### 2.1. Effect of In Vitro Digestion on α-Amylase Inhibitory Activity

Screening results of anti-α-amylase capacity of extracts from *Sargassum* spp. show that only hexane extract (NBH) displays inhibitory activity on α-amylase (IC_50_ = 0.963 mg/mL). In contrast, ethyl acetate/butanol and water extracts do not exhibit α-amylase inhibitory activity. Based on that, NBH was loaded into column chromatography to obtain three fractions (F1–F3). The inhibitory effect of in vitro gastrointestinal digestion on α-amylase inhibitory activity of these fractions and their dominant detected compound palmitic acid (PA) is presented in Table 1. In this assay, acarbose serves as a positive standard which displays the inhibition of α-amylase activity with an IC_50_ value of 0.06 mg/mL.

For undigested samples, the inhibition (IC_50_ value) of F1, F2, F3, and PA varies from 0.358 to 1.264 mg/mL. F1 presents the strongest prevention against α-amylase action with IC_50_ value of 0.358 mg/mL, followed by F3, F2, and PA with IC_50_ values of 0.430, 0.758, and 1.264 mg/mL, respectively (Table 1). Via in vitro oral digestion, both F1 and F2 increase the α-amylase inhibitory activity but F3 does not in relation to undigested fractions. In particular, oral digestion has a significant effect on F3 by reducing its α-amylase inhibition by 49.88% (IC_50_ = 0.858 mg/mL). On the other hand, the anti-α-amylase activity of PA raises up to 75.40% (IC_50_ = 0.311 mg/mL). At the end of oral phase, the activity order of tested samples is F1 and PA > F2 > F3. The α-amylase inhibition of F1 and F3 remarkably decreases over the digestion at the gastric phase, of which, the suppressive activity of F1 reduces 49.9% (IC_50_ = 0.715 mg/mL) and that of F3 declines 32.5% (IC_50_ = 0.637 mg/mL) in comparisons with those of undigested samples. Whereas the anti-α-amylase activity of F2 and palmitic acid increase 1.98% (IC_50_ = 0.743 mg/mL) and 58.07% (IC_50_ = 0.530 mg/mL), respectively. The IC_50_ value order can be found as PA > F3 > F1 and F2. In the intestinal phase, all fractions exhibit significant increases in α-amylase inhibitory ability except F3. In comparison with undigested samples, the activity of F1, F2, and PA increase by 13.97% (IC_50_ = 0.308 mg/mL), 44.95% (IC_50_ = 0.360 mg/mL), and 63.53% (IC_50_ = 0.461 mg/mL), respectively.

Although F3 shows a decrease in the activity with a loss of 15.35% (IC_50_ = 0.508 mg/mL) compared to that of undigested one, it significantly increases 1.25-fold compared to that in the gastric digestion. The order of α-amylase inhibition of tested samples at this phase is F1 > F2 > PA > F3.

In summary, the in vitro digestion significantly affects the α-amylase inhibitory activity of fractions isolated from NBH extract of *Sargassum* spp. Though all samples reduce the biological activity in the gastric phase, they have a significant increase in the intestinal phase. Additionally, all fractions perform remarkable activity over digestive stages compared with PA, a well-known α-amylase inhibitor, of which, the activity of F1 and F2 is 1.5- and 1.3-fold stronger than that of PA at the end of intestinal digestion. This suggests that hexane fractions of *Sargassum* spp. might be accessible via the digestive tract to become potential α-amylase inhibitors in the treatment of diabetes.

### 2.2. Effect of In Vitro Digestion on Cytotoxic Activity against U266 Cell Line

The cytotoxicity against U266 cell line of F1, F2, and F3 is significantly influenced by in vitro gastrointestinal digestion at *p* < 0.05, and the results are illustrated in Table 2.

For undigested samples, at the tested concentration of 0.2 mg/mL, F2 and F3 demonstrate the highest cytotoxic capacity against U266 cell line with inhibition percentages of 40.81% and 37.96%, respectively, followed by F1 (25.68%) and PA (24.65%). Whereas the standard anticancer agent doxorubicin shows outstanding cytotoxicity on U266 with an IC_50_ value of 0.13 µg/mL. 

Following the oral stage, the cytotoxic capacity of fraction F1 significantly increases by 19.66%, however, that of F2 and F3 reduces by 11.87% and 3.49%, respectively. Meanwhile, the antitumor activity of PA has no change compared to that of the undigested sample. The activity order among samples after the oral digestive stage is F2 and F3 > F1 > PA. In the gastric phase, fractions F2 and F3 continuously reduce the cytotoxicity against U266 cell line by 18.17% and 15.17%, respectively while F1 and PA significantly raise their activity by 26.50% and 39.90%, respectively. Nevertheless, all samples express the same biological activity which is in line with PA. At the end of the digestive process, intestinal phase, all samples significantly increase the antitumor effects compared to their undigested samples. Accordingly, the growth of U266 cell line is significantly inhibited by F2 (45.51%, rise of 11.53%), F3 (44.07%, rise of 16.09%), F1 (33.27%, rise of 29.55%), and PA (40.73%, rise of 65.25%) (Table 2). After the final stage, F2 and F3 exhibit the strongest activity which is in accordance with PA while F1 shows a weaker effect.

Over in vitro digestion, the cytotoxic activity of F1 progressively increases over the stages which are in line with that of PA. Besides, the biological activity of F2 and F3 is decreased over oral and gastric digestions, it is significantly enhanced at the end of the intestinal phase. This implies that hexane fractions isolated from *Sargassum* spp. could be absorbed and accessible via the digestive system as antitumor substances which might be prospectively applied in controlling the myeloma.

### 2.3. Identification and Quantification of Bioactive Compounds

Among extracts of *Sargassum* spp., hexane extract and the associated fractions F1, F2, F3 obtained from column chromatography over silica gel showed the most potent α-amylase inhibition and cytotoxicity. Therefore, they were selected to identify the bioactive compounds by using gas chromatography-mass spectrometry (GC-MS). 

The chemical components of hexane extract are shown in Table 3. Accordingly, ten major compounds are identified including myristic acid methyl ester (1.10%), myristic acid (3.79%), phytol acetate (4.29%), 6,10,14-trimethylpentadecan-2-one (1.48%), phytol (2.08%), palmitic acid methyl ester (18.65%), cis-4-tridecene (1.56%), palmitic acid (39.85%), palmitoleic acid (2.56%), and 2-palmitoylglycerol (3.37%). In which, palmitic acid and palmitic acid methyl ester are the most dominant constituents.

The main phytochemical constituents of F1, F2, and F3 are presented in Table 4. In F1, palmitic acid methyl ester is the most abundant compound (22.66%), followed by palmitic acid (13.14%), oleic acid methyl ester (2.68%), myristic acid methyl ester (1.66%), myristic acid (1.63%), 6,10,14-trimethylpentadecan-2-one (1.14%), and methyl palmitoleate (1.06%). Fucosterol (47.68%) is identified as a primary compound in F2, followed by palmitic acid (16.26%), desmosterol (4.08%), and myristic acid (1.85%). Similarly, the most abundant bioactive compound in F3 is fucosterol (46.15%), followed by desmosterol (11.94%), palmitic acid (8.88%), phytol (2.65%), and undecanoic acid (1.13%).

In this study, palmitic acid is identified in all fractions, of which, the compound is determined with the highest peak area at F2 (16.26%), followed by F1 (13.14%) and F3 (8.88%). The quantification of palmitic acid and fucosterol from these fractions using calibration curves of palmitic acid and fucosterol standards is illustrated in Table 5. F1 contains the highest amount of the palmitic acid compound (228.26 and 2.79 mg/g NBH and TDW, respectively), followed by F2 (34.75 and 0.43 mg/g NBH and TDW, respectively), and F3 (9.01 and 0.11 mg/g NBH and TDW, respectively). Fucosterol was detected in F2 with contents of 6.54 and 0.08 mg/g NBH and TDW, respectively. While in F3, fucosterol accounts for 2.68 and 0.03 mg/g NBH and TDW, respectively.

## 3. Discussion

The potential pharmaceutical properties of *Sargassum* spp. have been proven by recent advanced studies. However, investigations on the effect of the digestive system on such biological activities as anti-diabetes and anticancer have remained limited. This present study, for the first time, examined the effect in vitro digestion on α-amylase inhibitory and cytotoxicity on U266 cell line of *Sargassum* spp. extract. 

Basically, most of the α-amylase inhibitors from natural sources are extracted by polar solvents. In a study by Husni et al., ethyl acetate extract of *Sargassum hystrix* rich in phenolic compounds, performed potent α-amylase inhibitory activity [31]. In addition, fucoidan from *S. wightii* [32], polyphenols and fucoxanthin from *S. hemiphyllum* [33] extracted by polar solvents also indicated potential inhibition on α-amylase. However, in the present study, the bio-guided fractionation of *Sargassum* spp. methanolic extract showed that only one extract by hexane (a non-polar solvent) exhibited the potent activity against α-amylase while ethyl acetate/butanol and water extracts showed negligible effects. In fact, the distribution of bioactive compounds and their bioactivity depend on various factors including species, species habitats, preservation conditions of sample, drying and extraction methods, etc. Our findings suggest that the biological activity of *Sargassum* spp. collected in Cambodia may be mainly attributed to compounds included in hexane extract, therefore, this extract is selected as the target in this study.

Hexane is widely used for separating oil from plant materials in the edible oil industry. This solvent has a high affinity with nonpolar compounds such as most fatty acids, aliphatic hydrocarbons, linear terpenes, and some volatile compounds. According to GC-MS results, the dominant presence of fatty acids (palmitic acid and myristic acid), fatty acids methyl esters (FAMEs), and sterols (desmosterol and fucosterol) may contribute a role to anti-α-amylase activity of *Sargassum* spp. Preceding study indicated that the active site of key enzymes linked to type 2 diabetes may get blocked with fatty acid components, preventing the enzyme from binding to the substrate [34]. In addition, palmitic acid found in all fractions (F1–F3) was identified as a diabetic inhibitor by various earlier studies [35]. In this study, the α-amylase activity variations of F1 and F2 induced by in vitro digestion model were in line with those of palmitic acid (Table 1). Besides, fucosterol, a co-occurrent compound detected in F2 and F3, was reported as a diabetic inhibitor in other studies [36,37]. Orhan et al. reported palmitic acid and palmitic acid methyl ester could decrease the blood glucose level in diabetic rats [38]. Myristic acid was announced to improve hyperglycemia by decreasing insulin-responsive glucose levels in Nagoya-Shibata-Yasuda (NSY) mice [39]. In addition, phytol extracted from *Toona sinensis* leaves possessed antidiabetic activity [40]. Therefore, the high accumulation and the correlation among palmitic acid, fucosterol, FAMEs, and linear terpenes such as phytol might be exploited as a potential candidate for further studies of the antidiabetic property of *Sargassum* spp. 

In terms of cytotoxic activity, fractions F2 and F3 showed significant inhibition on the viability of U266 cells, which was in consistent with that of palmitic acid, a known anti-multiple myeloma molecule [41]. Additionally, the change in the cytotoxicity of F2 and F3 over gastric and intestinal phases in the simulated model was in line with that of palmitic acid. This is the first study reports the effect of in vitro digestion on the cytotoxicity of palmitic acid on U266 cell line. Along with palmitic acid, fucosterol, the major components of F2 and F3, was reported as a potent anticancer agent [42], of which, fucosterol significantly induced human promyelocytic leukemia HL-60 cells apoptosis [43]. Other compounds identified by GC-MS including desmosterol and phytol had potential anticancer properties [44,45]. Therefore, the cytotoxic ability of *Sargassum* spp. fractions might be principally attributed to the concomitant occurrence and the synergic interaction of palmitic acid, fucosterol, and other compounds (Table 4). 

Under the digestion process, the structure stability and functional group constituents could be changed which might result in the corresponding alterations in the biological activities of samples. In which, pH condition might be the main impact since most of the tested samples lowered their bioactivity under an acid pH at the gastric phase (Table 1 and Table 2). In fact, it is proven that the gastric stage degrades 30% of the vitamin C content in pomegranate juice compared to the initial sample [46]. At pH 3, the whey protein was digested more than that at pH 5 [47]. The results suggest that the various phenomena of biological activities of *Sargassum* spp. extracts might result from either probable formation or degradation of bioactive compounds or functional groups due to the pH shift in each digestive stage [48]. Though the effect of in vitro digestion process on biological activities of palmitic acid and fractions containing palmitic acid was elaborated in this study, it is necessary to evaluate the same effect on individual identified active compound such as fucosterol since this compound is a dominant compound in F2 and F3. 

Overall, the effects of digestion on variation of anti-amylase and cytotoxic activities of fractions from hexane extracts of *Sargassum* spp. may contribute to further studies on developing diabetes and cancer treatments. Besides, the chemical profiles of isolated fractions by GC-MS are prospective for future studies on bioaccessibility and bioavailability in the body. However, the identification and isolation of other α-amylase inhibitors in this *Sargassum* spp. by polar solvents should be carried out together with advanced spectroscopic techniques including LC-MS/MS and LC-HRMS to determine more active compounds with valuable medicinal and pharmaceutical activities from this seaweed. Furthermore, toxicology of these bioactive compounds as well as pharmacokinetic measurement, in vivo assays, and pre-clinical tests should be carried out to comprehensively validate the biological activities of target compounds. 

## 4. Materials and Methods

### 4.1. Chemicals and Equipment

α-Amylase porcine pancreas, soluble starch, silica gel, and buffer solution were purchased from Sigma-Aldrich, St. Louis, MO, USA. Acarbose and iodine solution were obtained from Fujifilm Wako Pure Chemical Corporation, Osaka, Japan. All organic extraction solvents were purchased from Junsei Chemical Co., Ltd., Tokyo, Japan. A Multiskan^TM^ microplate reader (Thermo Fisher Scientific Co., Ltd., Osaka, Japan), a Rotavapor^®^ R-300 (Nihon BUCHI K.K., Tokyo, Japan), JMS-T100 Gas Chromatography-Mass Spectrometer (JEOL Ltd., Tokyo, Japan), an incubator (Thermo Fisher Scientific, Waltham, MA, USA), and an inverted microscope (LabX, Midland, ON, Canada) were the main instruments used in the study. The U266 cell line (CVCL_0566) was obtained from American Type Culture Collection (ATCC), Virginia, USA.

### 4.2. Materials

**Figure 1:**
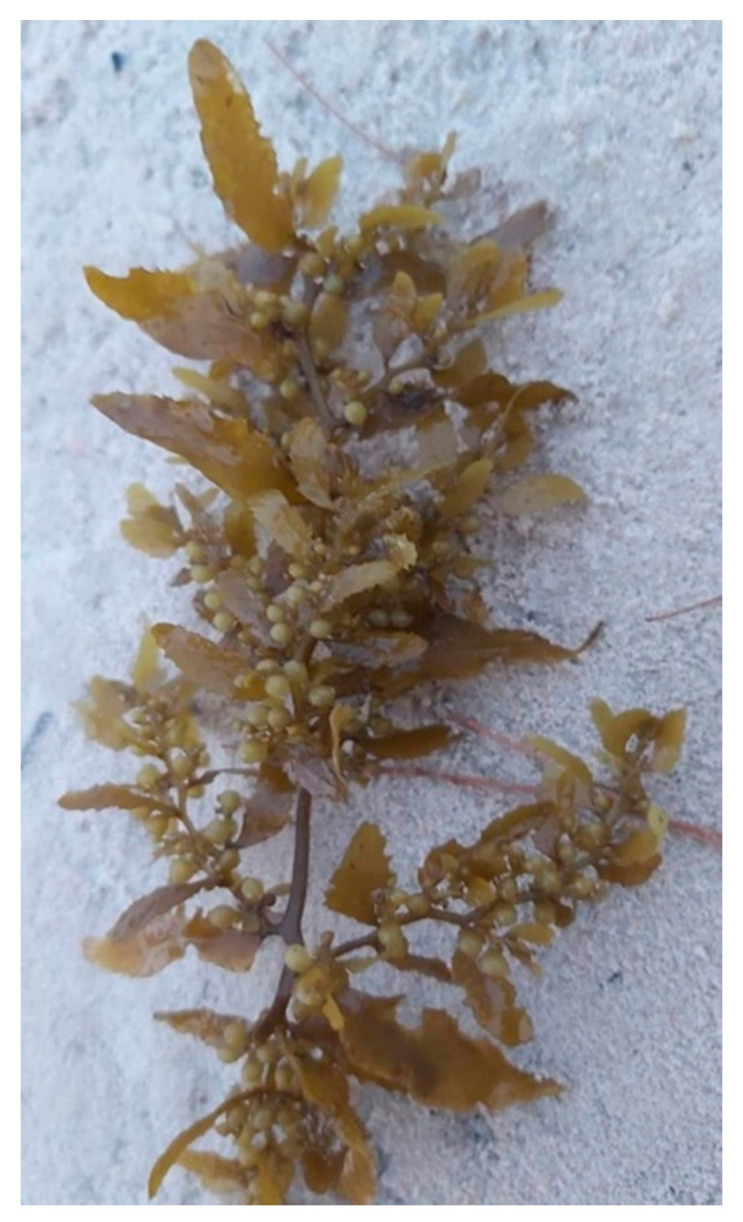
*Sargassum* spp. collected from Cambodia.

*Sargassum* spp. a sample including blade, meristem, and stipe was collected in Koh Ta Keav Island, Cambodia, in August 2019 (Figure 1). The seaweed was identified using morphological characteristics via open sources including AlgaeBase (www.algaebase.org) and BOLDSYSTEMS (http://www.boldsystems.org/, accessed on 30 August 2019). The specimen (NABA2019) was deposited at Agricultural Genetics Institute, Hanoi, Vietnam.

### 4.3. Extraction and Fractionation Process 

**Figure 2:**
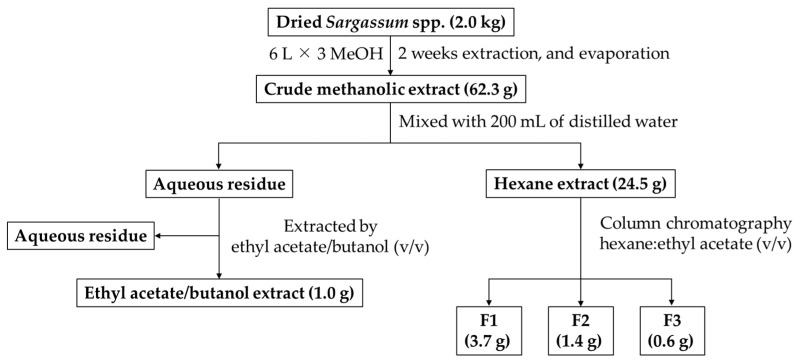
Extraction and isolation process of bioactive constituents from *Sargassum* spp.

Dried powder of *Sargassum* spp. (2 kg) was extracted three times with 6 L of methanol for two weeks at room temperature. After filtration, the methanolic extract was evaporated by a rotary evaporator to dryness under reduced pressure. The total crude methanolic extract weight (NBM) was 62.33 g. The NBM was mixed with 200 mL of distilled water to homogenize before fractionation. The NBM was fractionated by hexane and ethyl acetate-mix butanol. The obtained yield from hexane (NBH) and ethyl acetate/butanol (NBE) was 24.47 g and 0.96 g, respectively. All extracts and fractions were preliminarily screened for biological activities, afterwards, the potent active extracts or fractions were selected for further analyses. The process of extraction and isolation is presented in Figure 2.

The NBH extract was subjected to column chromatography over silica gel 60 (0.063–0.200 mm) (70–230 mesh ASTM), using hexane and ethyl acetate (from ratios of 10:0 to 3:7, *v*/*v*) as mobile phase. Particularly, each elution (50 mL/eluate) was separately collected and then tested the similarity by thin-layer chromatography (TLC). The discernment of compound separation of each fraction on TLC plate was visually detected after staining with 1% vanillin-sulfuric acid in pure ethanol and dried in an oven at 100 °C. Initially, the column was pre-washed with 500 mL of pure hexane. Subsequently, F1, F2, F3 fractions were successively collected from the hexane and ethyl acetate (9:1, *v*/*v*) elution, of which, F1 was obtained from eluates 1–6, F2 was a combination of eluates 7–8, and eluates 9–10 yielded F3. After combinations, the fractions were dried by a vacuum rotary evaporator at room temperature. The dry weight of each fraction was 3.72, 1.41, and 0.57 g for F1, F2, and F3, respectively. 

### 4.4. In Vitro Digestion Model

In vitro digestion model was conducted based on a reported method [49] with minor modifications. The model was designed into three phases including the mouth, stomach, and small intestine. The artificial saliva was designed for the oral digestive phase and was a mixture of 10 mL of KCl (89.6 g/L), 10 mL of KSCN (20 g/L), 10 mL of NaH_2_PO_4_ (88.8 g/L), 10 mL of Na_2_PO_4_ (57 g/L), 1.7 mL of NaCl (175.3 g/L), 8 mL of urea (25 g/L), 145 mg of α-amylase, 15 mg of uric acid, and 50 mg of mucin at pH 6.5 ± 0.1. The gastric juice was used in the gastric phase and was a combination of 15.7 mL of NaCl (175.3 g/L), 3 mL of NaH_2_PO_4_ (88.8 g/L), 9.2 mL of KCl (89.6 g/L), 18 mL of CaCl_2_ (22.2 g/L) × 2H_2_O, 10 mL of NH_4_Cl (30.6 g/L), 8.3 mL of HCl (37%), 10 mL of glucose (65 g/L), 10 mL of glucuronic acid (2 g/L), 3.4 mL of urea (25 g/L), 1 g of BSA, 1 g of pepsin, and 3 g of mucin at pH 1.0 ± 0.1. The intestinal juice was designed to use in the intestinal phase and was mixed from 40 mL of NaCl (175.3 g/L), 40 mL of NaHCO_3_ (84.7 g/L), 10 mL of KH_2_PO_4_ (8 g/L), 6.3 mL KCl (89.6 g/L), 10 mL of MgCl_2_ (5 g/L), 18 µL of HCl (37%), 4 mL of urea (25 g/L), 9 mL of CaCl_2_ × 2H_2_O (22.2 g/L), 1 g of BSA, 3 g of pancreatin, and 0.5 g of lipase at pH 7.8 ± 0.2.

The process is carried out by imitating the gastrointestinal physiology consisting of chemical components, stomach pH, temperature (37 °C), and transition time to determine the bioaccessibility of bioactive compounds. Particularly, at the beginning of the process, 2 mL of *Sargassum* spp. the sample was added to 3 mL of artificial saliva for 5 min in a shaking water bath at 37 °C. Then, 4 mL of gastric juice was added to the sample tube and the mixture was kept for 1 h at 37 °C. Finally, 4 mL of small intestinal juice was added to the sample tube and continued incubating for 1 h at 37 °C. Ethyl acetate was added into the sample tube (1:1, *v*/*v*) at the end of each digestive phase to reconstitute bioactive compounds. After vigorous mixing, the ethyl acetate phase was collected, evaporated, and prepared for biological assays, respectively. 

### 4.5. Porcine Pancreatic α-Amylase Inhibition Assay

The starch iodine method was conducted following the protocol improved by Quan et al. [50]. α-Amylase solution (20 U/mL) was freshly prepared in 0.2 M phosphate buffer (pH 6.9). Samples were prepared in dimethyl sulfoxide (DMSO) to make a stock solution (10 mg/mL). Iodine solution and starch were prepared in ionized water. A mixture of 40 µL of sample and the α-amylase solution was injected into a 96-well microplate and incubated for 10 min at 37 °C. Then, 30 µL of starch was injected into the mixture and incubated for 8 min at 37 °C. After that, 20 µL of 1 M hydrochloric acid was added to stop the reaction. Finally, 100 µL of iodine used as an indicator was pipetted. A microplate spectrophotometer was utilized to measure the reaction at the absorbance of 565 nm. Acarbose and palmitic acid (PA) were used as positive controls. The inhibitory percentage was calculated as follow:

% Inhibition = (AS − AC)/(AB − AC) × 100(1)

where, AS is the absorbance value of a sample solution, AB is the absorbance value of a solution without enzyme and starch, and AC is the absorbance value of a solution without a sample. The linear equation obtained from the dose-dependent curve was used to calculate the half inhibitory concentration (IC_50_). A lower IC_50_ value presents a stronger inhibitory effect.

### 4.6. Cytotoxic Activity against U266 Cell Line

The iodine,3-(4,5-dimethylthiazolyl)2,5-diphenyltetrazolium bromide (MTT) assay was applied in this study. Feta bovine serum (10%), L-glutamine (5 mM), Penicillin (100 IU/mL), and Streptomycin (100 µg/mL) were the substances of the Iscove’s Modified Dulbecco’s Medium (IMDM) cell culture media. Samples (0.2 mg/mL) were prepared in 0.1% DMSO (in culture media). The cells (5 × 10^3^) were seeded in 90 µL of culture media in each plate well and incubated at 37 °C for 24 h with CO_2_ 5%. Then, 10 µL of the sample were pipetted to each well. After 78 h, 10 µL of MTT solution (5 mg/mL) was supplied. The cells were continuously incubated at 37 °C for 4 h. After that, 100 µL of cell lysis buffer (10% SDS in 0.01 M HCl) was added to each well. An inverted microscope detected the cell expansion, and the absorbance of 595 nm was applied to determine the cell viability in value. The negative control was 10 µL of culture media solution instead of sample while palmitic acid and doxorubicin were used as positive ones. The inhibition percentage of a sample against U266 cell line was calculated as:

% Inhibition = (ABS_control_ − ABS_sample_)/ABS_control_ × 100 (2)

where ABS_control_ is absorbance of the reaction with negative control, and ABS_sample_ is the absorbance of reaction with sample.

### 4.7. Gas Chromatography-Mass Spectrometry Analysis 

The chemical analysis for the hexane extract and three fractions (F1, F2, F3) was carried out using a gas chromatography-mass spectrometry (GC-MS) system (JMS-T100 GCV, JEOL Ltd., Tokyo, Japan). Samples were prepared and automatically injected into the GC-MS system by an autosampler. Sample separations were accomplished using helium as carrier gas at a split ratio of 5:1. The beginning temperature started at 50 °C without holding, the temperature was reached 300 °C (10 °C/min) and maintained for 20 min. A capillary column (DB-5MS, 30 m × 0.25 mm I.D. × 0.25 µm film thickness) (Agilent Technologies, J&W Scientific Products, Folsom, CA, USA) was applied. The ionization voltage was 70 eV electron, and the mass scanning was ranged from 29 to 800 amu [51]. The chromatogram results and mass spectrum were compared to JEOL’s GC-MS Mass Center System Version 2.65a. 

Palmitic acid (PA) and fucosterol were pre-screened as major compounds in fractions. The contents of these constituents from *Sargassum* spp. were determined based on the calibration curve of PA and fucosterol standards. The results are expressed as mg/g of total sample dry weight. The quantities of these compounds were calculated following the equation:

(3) $$Q\;=\frac{A\;\times \;\mathrm{FW}}{\mathrm{DW}\;\times \;C}\%$$

where Q is quantity of palmitic or fucosterol from *Sargassum* spp. (mg/g), A is intensity value got from the calibration curve of PA or fucosterol standard (mg/mL), FW is a fraction weight (mg), DW is a sample weight (g), and C is a concentration of each fraction (mg/mL).

## 5. Conclusions

This study is the first to examine the influences of in vitro simulated digestive model on antidiabetic and cytotoxic potentials of *Sargassum* spp. All tested samples expressed remarkable α-amylase inhibitory and cytotoxic properties at the final stage of digestion. This implied that *Sargassum* spp. hexane fractions may be accessible for body absorption and probably be prospective for preventing diabetes and myeloma. The two major compounds palmitic acid and fucosterol may principally contribute to antidiabetic and cytotoxicity on human multiple myeloma U266 cell properties of fractions from *Sargassum* spp. Advanced spectroscopic techniques should be used to identify other potent bioactive compounds of the brown seaweed. Additionally, in vivo experiments and clinal trials should be further implemented to scrutinize the medicinal and pharmaceutical properties of active compounds from this natural source.

## Figures and Tables

**Table 1:** Effect of in vitro digestion on anti-α-amylase activity of palmitic acid, F1, F2, and F3 fractionated from *Sargassum* spp.

Sample	IC_50_ Value (mg/mL)				Variation (%)		
	U	O	G	I	O	G	I
F1	0.358 ± 0.006 ^b^	0.315 ± 0.004 ^c^	0.715 ± 0.013 ^a^	0.308 ± 0.005 ^c^	12.01	−49.93	13.97
F2	0.758 ± 0.010 ^a^	0.654 ± 0.014 ^b^	0.743 ± 0.034 ^a^	0.360 ± 0.007 ^c^	13.72	1.98	44.95
F3	0.430 ± 0.006 ^d^	0.858 ± 0.018 ^a^	0.637 ± 0.010 ^b^	0.508 ± 0.008 ^c^	−49.88	−32.50	−15.35
Palmitic acid	1.264 ± 0.110 ^a^	0.311 ± 0.015 ^c^	0.530 ± 0.004 ^b^	0.461 ± 0.019 ^b^	75.40	58.07	63.53

U, undigested sample; O, oral digestion; G, gastric digestion; I, intestinal digestion. Data expressed as the mean ± standard deviation (*n* = 3). Different letters in a row indicate significant differences by Tukey’s test (*p* ≤ 0.05).

**Table 2:** Effect of in vitro digestion on cytotoxic activity against U266 cell line of palmitic acid, F1, F2, and F3 fractionated from *Sargassum* spp. at a concentration of 0.2 mg/mL.

Sample	Inhibition (%)				Variation (%)		
	U	O	G	I	O	G	I
F1	25.68 ± 2.26 ^b^	30.73± 0.55 ^a^	32.49 ± 0.92 ^a^	33.27 ± 0.77 ^a^	19.69	26.50	29.55
F2	40.81 ± 1.01 ^b^	35.96 ± 1.54 ^c^	33.40 ± 1.84 ^c^	45.51 ± 0.87 ^a^	−11.87	−18.17	11.53
F3	37.96 ± 2.77 ^b^	36.64 ± 0.90 ^bc^	32.20 ± 1.66 ^c^	44.07 ± 1.52 ^a^	−3.49	−15.17	16.09
Palmitic acid	24.65 ± 2.75 ^c^	24.71 ± 1.48 ^c^	34.49 ± 4.89 ^b^	40.73 ± 2.75 ^a^	0.24	39.90	65.25

U, undigested sample; O, oral digestion; G, gastric digestion; I, intestinal digestion. Data are expressed as the mean ± standard deviation (*n* = 3). Different letters in a row indicate significant differences by Tukey’s test (*p* ≤ 0.05).

**Table 3:** Bioactive components of the hexane extract from *Sargassum* spp.

Identified Compounds	Chemical Classification	Formula	MW	RT	Peak Area (%)
Myristic acid methyl ester	Fatty acid methyl ester	C_15_H_30_O_2_	242	14.6	1.1
Myristic acid	Fatty acid	C_14_H_28_O_2_	228	14.97	3.79
Phytol acetate	Acyclic diterpenoids	C_22_H_42_O_2_	338	15.82	4.29
6,10,14-trimethylpentadecan-2-one	Sesquiterpenoids	C_18_H_36_O	268	15.87	1.48
Phytol	Diterpenoids	C_20_H_40_O	296	16.27	2.08
Palmitic acid methyl ester	Fatty acid methyl ester	C_17_H_34_O_2_	270	16.71	18.65
cis-4-Tridecene	Unsaturated hydrocarbons	C_13_H_26_	182	16.85	1.56
Palmitic acid	Fatty acid	C_16_H_32_O_2_	256	17.09	39.85
Palmitoleic acid	Fatty acid	C_16_H_30_O_2_	254	18.73	2.56
2-Palmitoylglycerol	Fatty acid ester	C_19_H_38_O_4_	330	21.89	3.37

MW, molecular weight; RT, retention time.

**Table 4:** Bioactive components of the fractions (F1, F2, and F3) from *Sargassum* spp.

No.	Identified Compounds	Chemical Classification	Formula	MW	Peak Area in Fractions (%)		
					F1	F2	F3
1	Myristic acid methyl ester	Fatty acid methyl esters	C_15_H_30_O_2_	242	1.66	-	-
2	Myristic acid	Fatty acids	C_14_H_28_O_2_	228	1.63	1.85	-
3	6,10,14-Trimethylpentadecan-2-one	Sesquiterpenoids	C_18_H_36_O	268	1.14	-	-
4	Palmitoleic acid methyl ester	Fatty acid methyl esters	C_17_H_32_O_2_	268	1.06	-	-
5	Palmitic acid methyl ester	Fatty acid methyl esters	C_17_H_34_O_2_	270	22.66	-	-
6	Palmitic acid	Fatty acids	C_16_H_32_O_2_	256	13.14	16.26	8.88
7	Oleic acid methyl ester	Fatty acid methyl esters	C_19_H_36_O_2_	296	2.68	-	-
8	Desmosterol	Sterols	C_27_H_44_O	384	-	4.08	11.97
9	Fucosterol	Sterols	C_29_H_48_O	412	-	47.68	46.15
10	Undecanoic acid	Fatty acids	C_11_H_22_O_2_	186	-	-	1.13
11	Phytol	Diterpenoids	C_20_H_40_O	296	-	-	2.65

MW: molecular weight.

**Table 5:** Quantification of palmitic acid and fucosterol by GC-MS in each fraction.

Fraction	Palmitic Acid (mg/g TDW)	Fucosterol (mg/g TDW)
F1	2.79	-
F2	0.43	0.08
F3	0.11	0.03

TDW, total dried weight; -, not determined.

## Data Availability

The data underlying this article are available in the article.

## Sample Availability

Samples of the compounds are available from the authors.

## References

[1] Saeedi P., Petersohn I., Salpea P., Malanda B., Karuranga S., Unwin N., Colagiuri S., Guariguata L., Motala A.A., Ogurtsova K. (2019). Global and regional diabetes prevalence estimates for 2019 and projections for 2030 and 2045: Results from the International Diabetes Federation Diabetes Atlas, 9th edition. Diabetes Res. Clin. Pract..

[2] Siddiqui A.A., Siddiqui S.A., Admad S., Siddiqui S., Adsan I., Sahu K. (2013). Diabetes: Mechanism, pathophysiology and management—A review. Int. J. Drug Dev..

[3] Tundis R., Loizzo M.R., Menichini F. (2010). Natural products as α-amylase and α-glucosidase inhibitors and their hypoglycaemic potential in the treatment of diabetes: An update. Mini-Rev. Med. Chem..

[4] Tarling C.A., Woods K., Zhang R., Brastianos H.C., Brayer G.D., Raymond J., Andersen D., Stephen G., Withers D. (2008). The search for novel human pancreatic α-amylase inhibitors: High-throughput screening of terrestrial and marine natural product extracts. ChemBioChem.

[5] Dimopoulos M., Terpos E. (2010). Multiple myeloma. Ann. Oncol..

[6] Gadó K., Pállinger E., Kovács P., Takács E., Szilvási I., Tóth B.E., Nagy G., Domján G., Falus A. (2002). Prolactin influences proliferation and apoptosis of a human IgE secreting myeloma cell line, U266. Immunol. Lett..

[7] Stattin P., Björ O., Ferrari P., Lukanova A., Lenner P., Lindahl B., Hallmans G., Kaaks R. (2007). Prospective study of hyperglycemia and cancer risk. Diabetes Care.

[8] Vigneri P., Frasca F., Sciacca L., Pandini G., Vigneri R. (2009). Diabetes and cancer. Endocr. Relat. Cancer.

[9] Ryu T.Y., Park J., Scherer P.E. (2014). Hyperglycemia as a risk factor for cancer progression. Diabetes Metab. J..

[10] Xu C.X., Zhu H.H., Zhu Y.M. (2014). Diabetes and cancer: Association, mechanisms, and implications for medical practice. World J. Diabetes.

[11] He X.-X., Tu S.M., Lee M.-H., Yeung S.-C.J. (2011). Thiazolidinediones and metformin associated with improved survival of diabetic prostate cancer patients. Ann. Oncol..

[12] Martin-Castillo B., Dorca J., Vazquez-Martin A., Oliveras-Ferraros C., Lopez-Bonet E., Garcia M., del Barco S., Menendez J.A. (2009). Incorporating the antidiabetic drug metformin in HER2-positive breast cancer treated with neo-adjuvant chemotherapy and trastuzumab: An ongoing clinical–translational research experience at the Catalan Institute of Oncology. Ann. Oncol..

[13] Libby G., Donnelly L.A., Donnan P.T., Alessi D.R., Morris A.D., Evans J.M.M. (2009). New users of metformin are at low risk of incident cancer: A cohort study among people with type 2 diabetes. Diabetes Care.

[14] Psarakis H.M. (2006). Clinical challenges in caring for patients with diabetes and cancer. Diabetes Spectr..

[15] Boisson-Vidal C., Haroun F., Ellouali M., Blondin C., Fischer A.M., De Agostini A., Borines M.G., De Leon R.L., Cuello J.L. (2013). Bioethanol production from the macroalgae *Sargassum* spp.. Bioresour. Technol..

[16] Liu L., Heinrich M., Myers S., Dworjanyn S.A. (2012). Towards a better understanding of medicinal uses of the brown seaweed *Sargassum* in traditional Chinese medicine: A phytochemical and pharmacological review. J. Ethnopharmacol..

[17] Motshakeri M., Ebrahimi M., Goh Y.-M., Othman H.H., Hair-Bejo M., Mohamed S. (2014). Effects of brown seaweed (*Sargassum polycystum*) extracts on kidney, liver, and pancreas of type 2 diabetic rat model. Evid. Based Complement. Altern. Med..

[18] Palanisamy S., Vinosha M., Manikandakrishnan M., Anjali R., Rajasekar P., Marudhupandi T., Manikandan R., Vaseeharan B., Prabhu N.M. (2018). Investigation of antioxidant and anticancer potential of fucoidan from *Sargassum polycystum*. Int. J. Biol. Macromol..

[19] Anjana A., Ahamed K.N., Ravichandiran V., Sumithra M., Anbu J. (2014). Anticancer activity of *Sargassum wightii* Greville on Dalton’s ascitic lymphoma. Chin. J. Nat. Med..

[20] Lailatussifa R., Husni A., Nugroho A.E. (2016). Anti-stress activity of *Sargassum polycystum* extracts using a cold restraint stress model. Food Sci. Biotechnol..

[21] Chong C.W., Hii S.L., Wong C.L. (2011). Antibacterial activity of *Sargassum polycystum* C. Agardh and *Padina australis* Hauck (Phaeophyceae). Afr. J. Biotechnol..

[22] Ningsih D.L.W., Trianto A., Widowati I., Magdugo R., Hurtado A., Marty C., Bourgougnon N. (2020). The potential of cytotoxin and antiviral in *Sargassum polycystum* and *Sargassum* ilicifolium’s polysaccharides extract. ILMU KELAUTAN: Indones. J. Mar. Sci..

[23] Saraswati, Giriwono P.E., Iskandriati D., Tan C.P., Andarwulan N. (2019). Sargassum seaweed as a source of anti-inflammatory substances and the potential insight of the tropical species: A review. Mar. Drugs.

[24] Nagappan H., Pee P.P., Kee S.H.Y., Ow J.T., Yan S.W., Chew L.Y., Kong K.W. (2017). Malaysian brown seaweeds *Sargassum siliquosum* and *Sargassum polycystum*: Low density lipoprotein (LDL) oxidation, angiotensin converting enzyme (ACE), α-amylase, and α-glucosidase inhibition activities. Food Res. Int..

[25] Ismail G.A., Gheda S.F., Abo-Shady A.M., Abdel-Karim O.H. (2020). In vitro potential activity of some seaweeds as antioxidants and inhibitors of diabetic enzymes. Food Sci. Technol..

[26] Narayani S.S., Saravanan S., Ravindran J., Ramasamy M.S., Chitra J. (2019). In vitro anticancer activity of fucoidan extracted from *Sargassum cinereum* against Caco-2 cells. Int. J. Biol. Macromol..

[27] Cilla A., González-Sarrías A., Tomas-Barberan F., Espín J.C., Barberá R. (2009). Availability of polyphenols in fruit beverages subjected to in vitro gastrointestinal digestion and their effects on proliferation, cell-cycle and apoptosis in human colon cancer Caco-2 cells. Food Chem..

[28] Krzyzanowska J., Czubacka A., Oleszek W. (2010). Dietary phytochemicals and human health. Adv. Exp. Med. Biol..

[29] Kong F., Singh R. (2008). A Model stomach system to investigate disintegration kinetics of solid foods during gastric digestion. J. Food Sci..

[30] Kong F., Singh R.P. (2008). Disintegration of solid foods in human stomach. J. Food Sci..

[31] Husni A., Sulistyo R.P., Rahma S.A., Nugraheni P.S., Budhiyanti S.A. (2020). In vitro antidiabetic activity of *Sargassum hystrix* extract and its etnyl acetate fractions. Sys. Rev. Pharm..

[32] Lakshmana Senthil S., Vinoth Kumar T., Geetharamani D., Suja G., Yesudas R., Chacko A. (2015). Fucoidan—An α-amylase inhibitor from *Sargassum wightii* with relevance to NIDDM. Int. J. Biol. Macromol..

[33] Hwang P.-A., Hung Y.-L., Tsai Y.-K., Chien S.-Y., Kong Z.-L. (2014). The brown seaweed *Sargassum hemiphyllum* exhibits α-amylase and α-glucosidase inhibitory activity and enhances insulin release in vitro. Cytotechnology.

[34] Su C.-H., Hsu C.-H., Ng L.-T. (2013). Inhibitory potential of fatty acids on key enzymes related to type 2 diabetes. BioFactors.

[35] Berraaouan A., Abid S., Bnouham M. (2013). Antidiabetic oils. Curr. Diabetes Rev..

[36] Gohari A.R., Payghami N., Jamili S., Rustaiyan A., Saeidnia S., Nikan M. (2015). Alpha-amylase inhibitory activity and sterol composition of the marine algae, *Sargassum glaucescens*. Pharmacogn. Res..

[37] Jung H.A., Islam N., Lee C.M., Oh S.H., Lee S., Jung J.H., Choi J.S. (2013). Kinetics and molecular docking studies of an anti-diabetic complication inhibitor fucosterol from edible brown algae *Eisenia bicyclis* and *Ecklonia stolonifera*. Chem. Interact..

[38] Orhan N., Aslan M., Demirci B., Ergun F. (2012). A bioactivity guided study on the antidiabetic activity of *Juniperus oxycedrus* subsp. *oxycedrus* L. leaves. J. Ethnopharmacol..

[39] Takato T., Iwata K., Murakami C., Wada Y., Sakane F. (2017). Chronic administration of myristic acid improves hyperglycaemia in the Nagoya–Shibata–Yasuda mouse model of congenital type 2 diabetes. Diabetologia.

[40] Hsieh T.-J., Tsai Y.-H., Liao M.-C., Du Y.-C., Lien P.-J., Sun C.-C., Chang F.-R., Wu Y.-C. (2012). Anti-diabetic properties of non-polar *Toona sinensis* Roem extract prepared by supercritical-CO_2_ fluid. Food Chem. Toxicol..

[41] Nagata Y., Ishizaki I., Waki M., Ide Y., Hossen A., Ohnishi K., Miyayama T., Setou M. (2015). Palmitic acid, verified by lipid profiling using secondary ion mass spectrometry, demonstrates anti-multiple myeloma activity. Leuk. Res..

[42] Mao Z., Shen X., Dong P., Liu G., Pan S., Sun X., Hu H., Pan L., Huang J. (2018). Fucosterol exerts antiproliferative effects on human lung cancer cells by inducing apoptosis, cell cycle arrest and targeting of Raf/MEK/ERK signalling pathway. Phytomedicine.

[43] Ji Y.-B., Ji C.-F., Yue L. (2014). Study on human promyelocytic leukemia HL-60 cells apoptosis induced by fucosterol. Bio-Med. Mater. Eng..

[44] Ho D.V., Hoang H.N.T., Vo H.Q., Nguyen H.M., Raal A., Nguyen H.T. (2018). A new triterpene ester and other chemical constituents from the aerial parts of *Anodendron paniculatum* and their cytotoxic activity. J. Asian Nat. Prod. Res..

[45] Pejin B., Kojic V., Bogdanovic G. (2014). An insight into the cytotoxic activity of phytol at in vitro conditions. Nat. Prod. Res..

[46] Pérez-Vicente A., Gil-Izquierdo A., García-Viguera C. (2002). In vitro gastrointestinal digestion study of pomegranate juice phenolic compounds, anthocyanins, and vitamin C. J. Agric. Food Chem..

[47] Hodgkinson A.J., Wallace O.A., Boggs I., Broadhurst M., Prosser C.G. (2018). Gastric digestion of cow and goat milk: Impact of infant and young child in vitro digestion conditions. Food Chem..

[48] Wootton-Beard P., Moran A., Ryan L. (2011). Stability of the total antioxidant capacity and total polyphenol content of 23 commercially available vegetable juices before and after in vitro digestion measured by FRAP, DPPH, ABTS and Folin–Ciocalteu methods. Food Res. Int..

[49] Gutiérrez-Grijalva E.P., Angulo-Escalante M.A., León-Félix J., Heredia J.B. (2017). Effect of in vitro digestion on the total antioxidant capacity and phenolic content of 3 species of Oregano (*Hedeoma patens*, *Lippia graveolens*, *Lippia palmeri*). J. Food Sci..

[50] Quan N.V., Xuan T.D., Anh L.H., Tran H.-D. (2019). Bio-guided isolation of prospective bioactive constituents from roots of *Clausena indica* (Dalzell) Oliv. Molecules.

[51] Quan N.V., Xuan T.D., Tran H.-D., Thuy N.T.D., Trang L.T., Huong C.T., Andriana Y., Tuyen P.T. (2019). Antioxidant, α-amylase and α-glucosidase inhibitory activities and potential constituents of *Canarium tramdenum* Bark. Molecules.

